# Notch-based gene signature for predicting the response to neoadjuvant chemotherapy in triple-negative breast cancer

**DOI:** 10.1186/s12967-023-04713-3

**Published:** 2023-11-15

**Authors:** Mohamed Omar, Pier Vitale Nuzzo, Francesco Ravera, Sara Bleve, Giuseppe Nicolò Fanelli, Claudio Zanettini, Itzel Valencia, Luigi Marchionni

**Affiliations:** 1 Department of Pathology and Laboratory Medicine, Weill Cornell Medicine, New York, NY USA; 2 Dana Farber Cancer Institute, Boston, MA USA; 3 Department of Internal Medicine, University of Genoa, Genoa, Italy; 4 Department of Medical Oncology, IRCCS Istituto Romagnolo per lo Studio dei Tumori (IRST) “Dino Amadori”, Meldola, Italy; 5 First Division of Pathology, Department of Translational Research and New Technologies in Medicine and Surgery, University of Pisa, 56126 Pisa, Italy

**Keywords:** Triple negative breast cancer, Neoadjuvant chemotherapy, Gene signature, Notch signaling pathway, Prediction of therapeutic response

## Abstract

**Background:**

While the efficacy of neoadjuvant chemotherapy (NACT) in treating triple-negative breast cancer (TNBC) is generally accepted, not all patients derive benefit from this preoperative treatment. Presently, there are no validated biomarkers to predict the NACT response, and previous attempts to develop predictive classifiers based on gene expression data have not demonstrated clinical utility. However, predictive models incorporating biological constraints have shown increased robustness and improved performance compared to agnostic classifiers.

**Methods:**

We used the preoperative transcriptomic profiles from 298 patients with TNBC to train and test a rank-based classifier, k-top scoring pairs, to predict whether the patient will have pathological complete response (pCR) or residual disease (RD) following NACT. To reduce overfitting and enhance the signature’s interpretability, we constrained the training process to genes involved in the Notch signaling pathway. Subsequently, we evaluated the signature performance on two independent cohorts with 75 and 71 patients. Finally, we assessed the prognostic value of the signature by examining its association with relapse-free survival (RFS) using Kaplan‒Meier (KM) survival estimates and a multivariate Cox proportional hazards model.

**Results:**

The final signature consists of five gene pairs, whose relative ordering can be predictive of the NACT response. The signature has a robust performance at predicting pCR in TNBC patients with an area under the ROC curve (AUC) of 0.76 and 0.85 in the first and second testing cohorts, respectively, outperforming other gene signatures developed for the same purpose. Additionally, the signature was significantly associated with RFS in an independent TNBC patient cohort even after adjusting for T stage, patient age at the time of diagnosis, type of breast surgery, and menopausal status.

**Conclusion:**

We introduce a robust gene signature to predict pathological complete response (pCR) in patients with TNBC. This signature applies easily interpretable, rank-based decision rules to genes regulated by the Notch signaling pathway, a known determinant in breast cancer chemoresistance. The robust predictive and prognostic performance of the signature make it a strong candidate for clinical implementation, aiding in the stratification of TNBC patients undergoing NACT.

**Supplementary Information:**

The online version contains supplementary material available at 10.1186/s12967-023-04713-3.

## Background

Breast cancer (BC) is the most common non-cutaneous cancer, affecting over 2 million women worldwide and resulting in more than 600,000 deaths annually [1]. Triple-negative breast cancer (TNBC), which is characterized by the lack of expression of estrogen receptor (*ER*), progesterone receptor (*PR*), and epidermal growth factor receptor 2 (*EGFR2/HER2*), represents 10–20% of total BC cases and accounts for approximately 30% of BC-related deaths [2]. Compared to the other molecular subtypes of BC, TNBC is more aggressive and is more common among African-American women and in women below the age of 40 [3–5].

Neoadjuvant chemotherapy (NACT) represents the standard treatment for both locally advanced and early-stage TNBC in cases of tumor radiological diameter ≥ 1 cm and/or evidence of nodal dissemination [6]. The role of NACT is manifold, as it brings to tumor downstaging, allowing surgery to be performed on otherwise unresectable cancers or obtaining a better cosmetic outcome in patients with a high tumor-to-breast ratio, and provides useful prognostic biomarkers for patients’ postsurgical management [6].

The optimal achievement of NACT is pathological complete response (pCR), defined according to the ctNeoBC criteria as the absence of invasive BC in the breast and the axillary nodes (ypT0 ypN0 or ypT0/is ypN0) [7]. Recent studies indicate that the addition of platinum and/or immune checkpoint inhibitors to standard regimens based on anthracyclines and taxanes is associated with an increased probability of pCR for TNBC, with rates up to 55% [6]. Despite such results, 45–55% of TNBC patients present with residual disease (RD) at surgery, leading to significantly worse event-free survival and overall survival rates compared to patients achieving pCR [6, 8]. Moreover, NACT is associated with various toxicities, including gastrointestinal side effects and myelotoxicity, necessitating a comprehensive evaluation of patients prior to initiating therapy.

In this context, the development of a predictive classifier capable of stratifying patients based on their response to NACT is of paramount interest. Such a tool would optimize therapeutic decisions in the neoadjuvant setting, avoiding exposing patients classified as non-responders to the side effects of chemotherapy while retaining the prognostic information related to their treatment response, which is essential for their postsurgical management.

Numerous studies have attempted to identify gene expression-based signatures to predict the response to NACT in BC patients. However, these studies face significant limitations. For instance, many do not include patients with TNBC or comprise highly heterogeneous populations in terms of tumor subtypes. Studies focusing on TNBC often have small sample sizes or propose signatures that are challenging to implement in clinical practice owing to the large number of genes involved. Furthermore, these studies typically use agnostic approaches for developing gene signatures, resulting in less robustness compared to classifiers built upon biologically consistent constraints [9].

In this study, we present a gene signature designed to predict pCR in patients with TNBC. This signature was developed using a rank-based machine learning algorithm applied to gene expression datasets of TNBC patients, with a specific focus on genes associated with the Notch signaling pathway. The Notch signaling pathway plays a pivotal role in maintaining cancer stem cells and mediating tumor progression and chemoresistance in various types of cancer, including TNBC [10, 11]. Hence, it was selected as a biological constraint to enhance the robustness and the overall performance of our signature. The resulting classifier was evaluated in two independent test cohorts, displaying high and consistent accuracy. Importantly, our signature is specific to the triple-negative subtype, outperforms other signatures in predicting pCR, and retains prognostic information independent of the administration of NACT.

## Methods

### Data collection and inclusion criteria

We searched the Gene Expression Omnibus (GEO) for gene expression datasets using the following search terms: ((Breast cancer) AND chemotherapy) AND "Homo sapiens"[porgn: txid9606], applying the filters: entry type: series, study type: gene expression by array, and attribute name: tissue. The inclusion criteria consisted of breast fine needle aspiration (FNA) or core biopsy samples obtained from patients with TNBC before initiating NACT, with information about the pathologic response.

### Data preprocessing

In our preprocessing steps, we ensured that all datasets were normalized and log-scaled before analysis, and we Z-transformed the gene expression of each dataset separately to ensure that all datasets were on the same scale. Subsequently, we combined a portion of the datasets together in a single metadata based on a subset of common genes. We then partitioned the data into a training set and a testing set (first testing dataset) comprising 70% and 30% of the samples, respectively. By using balanced stratification, we ensured that both divisions had an equal representation of important covariates, such as age, tumor grade, T-stage, N-stage, and the parent dataset. An additional dataset was used for an independent assessment of the signature performance as a second testing dataset (Additional file 1: Table S1).

### Construction of the Notch mechanism

Well-established biological knowledge supports the role of the Notch signaling pathway in mediating cancer stem cell plasticity and chemoresistance in several cancer types, including BC [12]. To improve the performance and robustness of our classifier, we embedded this preexisting knowledge into its decision rules by building a set of gene pairs that captured Notch signaling biology. Specifically, we used the Molecular Signature Database (MSigDB) [13] to retrieve gene sets comprising genes regulated by Notch signaling and extracted their individual genes. Subsequently, we paired genes upregulated with those downregulated by NOTCH signaling to build a matrix of gene pairs, each consisting of a gene upregulated and another downregulated by Notch signaling. This matrix was then used as biological constraints during the training of the k-top scoring pairs (k-TSPs) algorithm as described below.

### Training the k-top scoring pairs model

The k-TSPs algorithm was used to identify gene pairs whose relative ordering consistently switched between the two classes of interest, namely, pCR versus RD. To reduce noise and identify informative features, we imposed biological constraints on the model training process by restricting the pair search process to the Notch mechanism described above. Subsequently, the k-TSPs algorithm was employed to identify all possible gene pairs whose expression consistently changed in samples from patients who achieved pCR and those who had RD after NACT. These pairs were further refined using a robust feature selection process to select the smallest set of pairs that could distinguish between these two phenotypes.

### Feature selection

We performed a feature selection process using a regularized random forest (RRF) [14, 15] on the training data. The RRF algorithm is similar to random forest (RF) but adds a penalty on the features used for splitting if their information gain is similar to features used at previous splits [14, 15]^.^ We bootstrapped the training data 100 times, and we trained an RRF model on each. To control overfitting, we used a regularization coefficient of 0.5 and an initial feature set of 0.1. To account for the imbalance in class size, we used prior weights of 1.0 and 0.6 for pCR and RD, respectively. Given that this training process is repeated on 100 different resamples of the training data, it is expected that the selected features would be different with each training round; however, important features should be selected more frequently. We ranked the selected features based on their frequency across the 100 iterations and selected the most frequent gene pairs to be our final classifier. It is important to note that while RRF was used for feature selection, the final classifier is still rank-based, and its decision rules follow those of the k-TSPs algorithm [16, 17].

### Evaluation of performance

We evaluated the resulting k-TSPs signature on the testing data using different performance metrics, including the area under the receiver operating characteristic (ROC) curve (AUC), balanced accuracy, sensitivity, specificity, and Matthews correlation coefficient (MCC). In addition to the testing set generated using the stratified sampling approach, we used an additional dataset [18, 19] for extra validation of the signature performance. Notably, p-values for the AUC-ROC curves were derived from the Delong’s test [20] comparing the classifier’s ROC-AUC to a random ROC curve with AUC of 0.5. Similarly, we assessed the performance of our signature against other signatures using Delong’s method, specifically testing if our signature’s AUC was significantly higher.

### Testing the signature on non-triple-negative breast cancer samples

To test the specificity of the signature, we used it to distinguish RD from pCR in all non-TNBC samples extracted from the same datasets used for the training and testing process. The prediction probability scores were then used to plot the ROC curve and calculate the AUC.

### Comparison with existing signatures in predicting the response to NACT

To further assess the predictive power and potential clinical utility of our signature, we compared the signature’s performance at distinguishing RD and pCR with other signatures available from similar studies. We queried the literature and identified several studies introducing gene signatures that are either predictive of the response to NACT in BC patients or based on signaling pathways and processes related to the chemotherapeutic response [21]. For instance, Zhao et al. developed a gene signature based on the expression of 143 genes and used it to develop a probability score for response to NACT in patients with TNBC [22]. Other signatures included in this analysis included signatures associated with acidosis response [23], androgen receptor (AR) signaling, epithelial-mesenchymal transition (EMT), growth factors, stemness, proliferation, DNA damage, and WNT signaling [24] together with other signatures associated with response to taxane therapy in TNBC [25]. All signatures were trained and tested on the same training and testing sets, respectively, to provide a fair assessment of performance. Genes from each signature were compiled into a logistic regression model to predict the response to NACT (pCR versus RD), and their testing performance was compared using the AUC metric.

### Association of the gene signature with recurrence-free survival

We then proceeded to test whether the signature was also associated with RFS in TNBC patients who received adjuvant chemotherapy. For this purpose, we used the Molecular Taxonomy of Breast Cancer International Consortium (METABRIC) cohort [26]. First, we computed the probability of RD in each sample based on the gene signature and then categorized this probability into low and high scores. These were then used to compute the RFS over time using Kaplan‒Meier survival estimates [27]. Furthermore, we performed a multivariate Cox proportional hazard model [28] accounting for other clinical and pathological variables, including T stage, age, type of breast surgery, menopausal status, and radiotherapy administration.

### Software and packages

All steps of this analysis were performed using R version 4.0.3. The feature selection process was performed using the RRF and boot packages [14, 15, 29], while the k-TSPs model was trained using the SwitchBox R package [30]. Finally, survival analysis was performed using the survival [31, 32] and survminer [33] packages.

## Results

### Data collection and study population

Our search in the GEO portal initially identified 98 gene expression datasets. Of these, seven met our inclusion criteria. A total of 369 TNBC samples collected before treatment initiation were selected and included in subsequent analysis (see Additional file 1: Table S1). Samples from six of the seven datasets were integrated together after appropriate normalization into metadata of 298 samples and used as a training set and as a first testing set, including 70% and 30% of the samples, respectively. The 7th dataset, including 71 samples, was used as a second testing cohort. Notably, the training and testing cohorts had a similar representation of relevant variables, including age, tumor grade, and stage (Table 1).

**Table 1:** Characteristics of the datasets used in the analysis

	Training	Testing 1	Testing 2
Number of samples	223 (RD = 150, pCR = 73)	75 (RD = 50, pCR = 25)	71 (RD = 46, pCR = 25)
Mean Age in years (± SD)	49.9 (± 10.9)	50.3 (± 11)	50.4 (± 10.8)
Grade			
Grade 1	2 (1%)	1 (1%)	0 (0%)
Grade 2	32 (14%)	15 (20%)	11 (15.5%)
Grade 3	153 (69%)	44 (59%)	53 (74.6%)
Unknown	36 (16%)	15 (20%)	7 (9.9%)
T-stage			
T1	10 (4%)	2 (3%)	6 (8.5%)
T2	98 (44%)	40 (53%)	33 (46.5%)
T3	59 (27%)	15 (20%)	14 (19.7%)
T4	42 (19%)	11 (15%)	17 (23.9%)
Unknown	14 (6%)	7 (9%)	1 (1.4%)
N-stage			
N0	52 (23%)	20 (27%)	11 (15.5%)
N1	86 (39%)	24 (32%)	36 (50.7%)
N2	33 (15%)	10 (13%)	13 (18.3%)
N3	21 (9%)	4 (5%)	10 (14.1%)
Unknown	30 (14%)	17 (23%)	1 (1.4%)

The training data were used to develop the classifier, and the two testing datasets were used to evaluate its performance. *RD* residual disease, *pCR* pathological complete response

### Construction of the Notch mechanism

Using the molecular signature database [13], we identified 16 gene sets comprising genes that are up- (13 gene sets, comprising 203 genes) or down-regulated (3 gene sets, comprising 134 genes) by NOTCH signaling. The genes that are present in both up-and down-regulated gene sets were filtered out yielding a total of 189 and 120 genes that are up- and down-regulated by Notch signaling. Each up-regulated gene was paired with each down-regulated gene to build the Notch mechanism as the following:

$$Number\,of\,pairs=189\times 120$$

This resulted in a total of 22,680 gene pairs. Genes that are missing in the expression matrices of the training and testing cohorts were then filtered out, setting the final number of gene pairs to 17,898 pairs (see Additional file 2).

### Five gene pairs predict the response to neoadjuvant chemotherapy before treatment

**Fig. 1:**
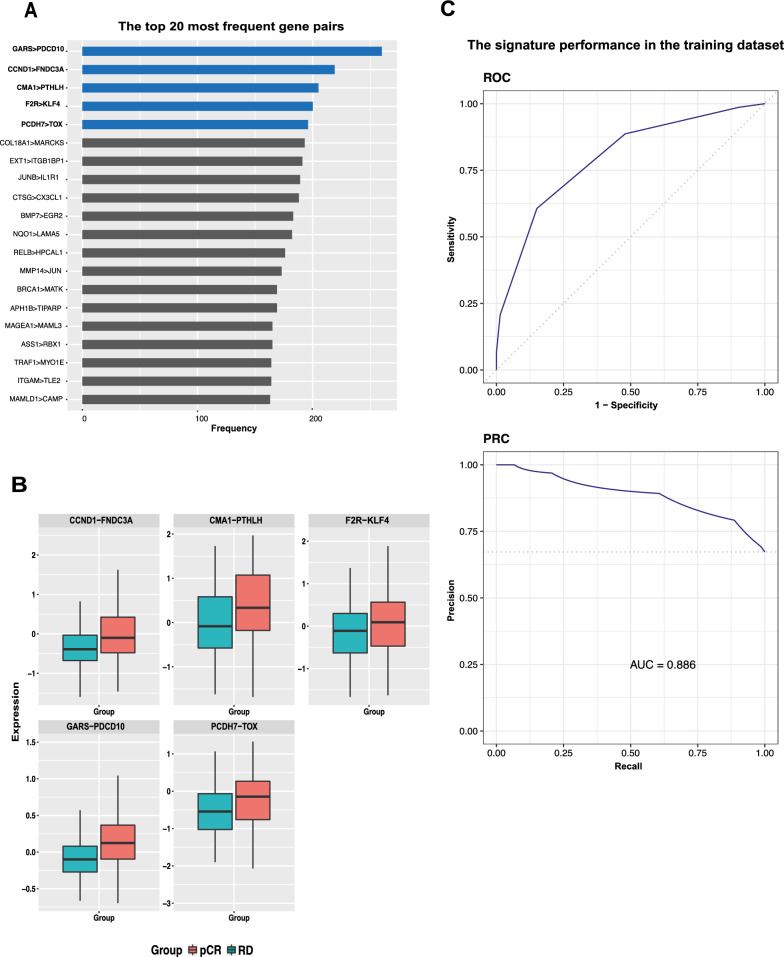
Discovery of the Notch 5-TSPs signature. **A** Selection frequency of the top 20 gene pairs. Regularized random forest was run 100 times on bootstrapped training data to select the most informative features from the list of 114 Notch-based TSPs returned by the k-TSPs algorithm. The 5 most frequently selected pairs were used in the final classifier (5-TSPs). **B** Boxplots showing the relative expression of each pair in resistant (RD) versus sensitive (pCR) samples. **C** Receiver operating characteristic (ROC) (top) and precision-recall (PRC) (bottom) curves showing the performance of the Notch 5-TSPs signature in predicting the response to NACT in the training data

Using the a priori mechanism representing Notch signaling, we trained a k-TSPs classifier to return the maximum number of pairs that consistently switched their expression ranking between RD and pCR. With this mechanistic process, we identified 114 top-scoring pairs (TSPs), whose relative ordering was consistently switched between samples with pCR and RD, with each consisting of a gene that is up- and down-regulated by Notch signaling (see Additional file 3). Subsequently, we used another feature selection algorithm (RRF) to select the smallest subset of pairs that could distinguish RD from pCR. Specifically, we trained RRF models on 100 bootstraps of the training data and ranked the returned features (gene pairs) based on their frequency across the 100 iterations (see Fig. 1A). Finally, the top five most frequent gene pairs were selected as the final signature and were used in all downstream analyses (henceforth referred to as the Notch 5-TSPs signature). This signature includes *GARS* > *PDCD10*, *CCND1* > *FNDC3A*, *CMA1* > *PTHLH*, *F2R* > *KLF4*, and *PCDH7* > *TOX.* Each pair votes for “RD” if the first gene is overexpressed relative to the second, and the final prediction is based on the sum of votes using a threshold of two votes for “RD” class prediction (see Fig. 1B). These simple decision rules could effectively distinguish RD from pCR in the training data with areas under the receiver operating characteristic curve (ROC) and precision-recall curve (PRC) of 0.80 and 0.89, respectively (Fig. 1C).

### The Notch 5-TSPs signature is predictive of the NACT response in two separate testing sets

**Fig. 2:**
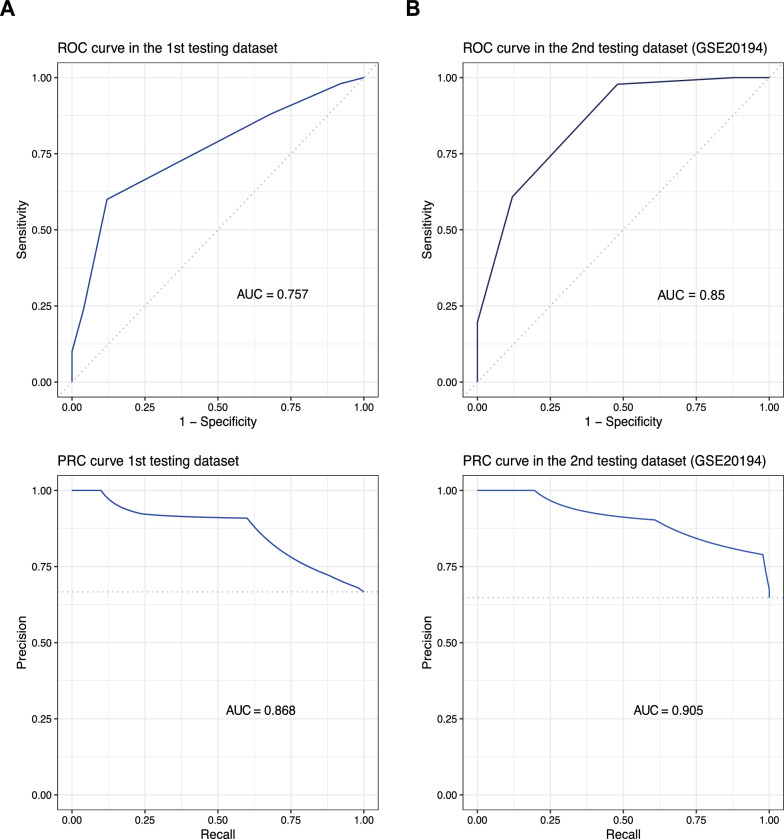
Performance of the Notch 5-TSPs signature in the testing sets. The signature includes five gene pairs that distinguish RD from pCR before initiating NACT in the two testing datasets (**A**, **B**). ROC (upper panel) and PRC (lower panel) curves were used to evaluate the performance. *ROC* Receiver operating characteristic curve. *PRC* Precision Recall Curve. *AUC* Area under the ROC curve. *AUPRC* Area Under the Precision Recall Curve

To assess the performance of the signature, we tested its predictive performance in pretreatment samples from two separate patient cohorts not used for its training. These testing sets included samples from 75 and 71 patients. In both sets, the Notch 5-TSPs signature could effectively distinguish RD from pCR with AUCs of 0.76 (95% CI: 0.65–0.86, p-value = 8.3e-07) and 0.85 (95% CI: 0.76–0.94, p-value = 1.7e-11), respectively (Fig. 2). Using a threshold of two votes to predict RD, the signature could correctly predict 44 out of 50 RD events (sensitivity = 88%) in the first testing set. In the second testing set, the signature correctly predicted 45 out of 46 RD events (sensitivity = 98%).

### The Notch 5-TSPs signature is specific to the triple negative phenotype

**Fig. 3:**
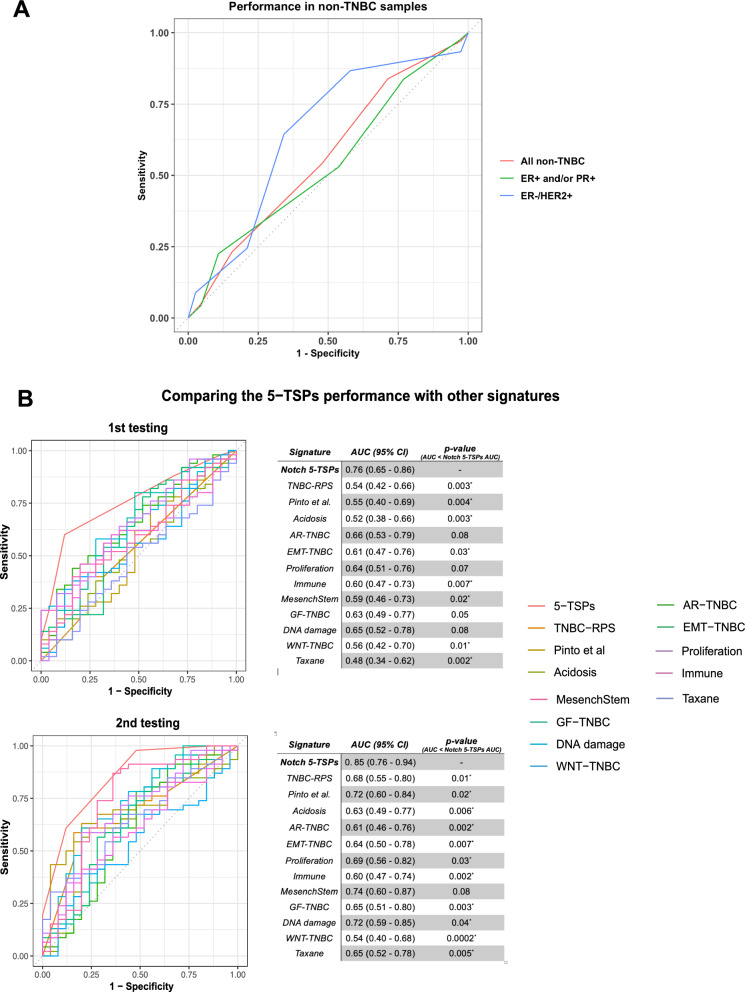
The Notch 5-TSPs signature performance in non-TNBC samples was compared with similar signatures. **A** The Notch 5-TSPs signature was evaluated in all non-TNBC, ER/PR + , and ER-/HER2 + samples. **B** Receiver operating characteristic (ROC) curves comparing the performance of the Notch 5-TSPs signature in predicting the response to NACT in TNBC with similar signatures in the two testing sets. Each table displays the Area under the ROC curve (AUC), 95% confidence interval (CI), and p-value from Delong’s test, testing the hypothesis that the AUC of the Notch 5-TSPs signature is greater than each respective signature’s AUC. *TSPs* top-scoring pairs. *TNBC* triple-negative breast cancer, *AR* androgen receptor, *EMT* epithelial-mesenchymal transition, *MesenchStem* mesenchymal stem-like, *GF* growth factor

Subsequently, we examined the signature performance on the remaining non-TNBC samples collected from the datasets used for the training and testing processes. This sample set included a total of 844 BC cases, of which 720 are ER/PR + BCs and 83 are ER-/HER2 + BCs. Notably, the signature had a weak performance for all non-TNBC samples and for the ER/PR + subset, with AUCs of 0.56 and 0.54, respectively. However, the performance was better in the ER-/HER2 + subset, with an AUC of 0.65 (Fig. 3A).

### The Notch 5-TSPs signature outperforms other signatures in predicting the response to NACT in patients with TNBC

Over the past years, several gene signatures have been introduced to predict the response to NACT and other important phenotypes in patients with BC [21, 22]. We retrieved the genes comprising each signature, including our Notch 5-TSPs signature, and used their expression values to train a logistic regression model using our training set described above to predict the response to NACT in patients with TNBC. To ensure a fair assessment of performance, we used logistic regression as a unified algorithm to re-train all signatures on the same training data and then evaluated their performances on both our testing sets. Notably, the Notch 5-TSPs signature had a higher AUC than all the other 12 signatures in both testing sets (Fig. 3B). Furthermore, we tested if the AUC of our Notch 5-TSPs signature is significantly higher than the AUCs of other signatures using the Delong’s test. We found that our signature’s AUC is significantly higher than 8 and 11 out of 12 signatures in the 1^st^ and 2^nd^ testing cohorts, respectively (Fig. 3B). In addition to its superior performance, these results also highlight the robustness of our signature even when trained using a different ML algorithm (logistic regression versus k-TSPs) whose decision rules are significantly different.

### The Notch 5-TSPs signature assessed in surgical samples after adjuvant chemotherapy captures prognostic information in patients with TNBC

RD after NACT is associated with worse outcomes, especially in patients with TNBC. With this in mind, we proceeded to test whether our signature was associated with RFS in a subset of the METABRIC dataset including samples from 198 patients with TNBC who received adjuvant chemotherapy. First, we tested the predictive performance of the 5-TSPs signature in this patient cohort by using it to distinguish patients who relapsed (RFS = 1) from those who did not (RFS = 0). Interestingly, while our signature was trained on pretreatment samples to predict the response to NACT, it also had a good performance at predicting RFS in posttreatment surgical samples (Additional file 1: Fig. S1A). Subsequently, we used Kaplan‒Meier survival analysis to assess the prognostic value of the signature and found that patients predicted to relapse (RFS = 1) had a significantly lower RFS than those predicted to be non-relapsing (RFS = 0) (Additional file 1: Fig. S1B). Of the five gene pairs comprising the Notch 5-TSPs signature, four were also present in the METABRIC dataset. We tested whether each individual pair could also capture RFS based on the pairwise ranking of its two genes. One pair (*CCND1* > *FNDC3A*) had a significant association with RFS, with samples in which *CCND1* was overexpressed relative to *FNDC3A* having a less favorable PFS (log-rank p value = 0.00036) (Additional file 1: Fig. S1C). The remaining three pairs showed a similar pattern but without statistical significance.

**Fig. 4:**
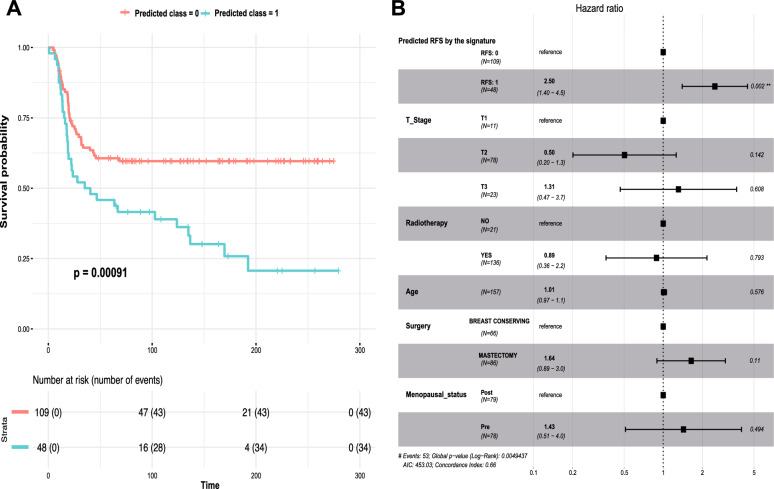
The Notch 5-TSPs signature is significantly associated with relapse-free survival in patients with TNBC within the METABRIC dataset. **A** Kaplan‒Meier survival analysis showing the association between the signature’s predictions and relapse-free survival in 157 patients with TNBC included in the METABRIC dataset. **B** Forest plot showing the results of multivariate survival analysis using the Cox proportional hazards model. The multivariate Cox model included the signature’s predictions together with T-stage, radiotherapy, age, type of surgery, and menopausal status. The hazard ratios and 95% confidence intervals are displayed. * denotes statistical significance

Similar to our rank-based 5-TSPs signature, the signature trained using logistic regression (see “Methods” section) was also significantly associated with RFS in the METABRIC dataset using univariate Kaplan‒Meier survival analysis (Fig. 4A). Additionally, we performed a multivariate survival analysis using a Cox regression hazards model adjusting for important clinical covariates, including T-stage, radiotherapy, age, type of breast surgery (breast-conserving surgery versus mastectomy), and menopausal status (pre- and post-menopause). The 5-TSPs signature was still significantly associated with worse RFS (HR = 2.5, 95% CI = 1.4–4.5) independent of other variables (Fig. 4B).

## Discussion

The development of accurate predictors of the response to chemotherapy could revolutionize the management of patients with early BC who are suitable to receive NACT [8, 34, 35]. Patients identified as non-responders could potentially avoid NACT and its associated side effects while retaining valuable prognostic information tied to therapeutic response. Simultaneously, trials of treatment escalation could be envisioned to improve their short- or long-term outcomes.

Several studies have endeavored to identify gene expression-based signatures predictive of pCR after NACT in BC patients. For instance, Hatzis et al. developed two gene signatures for pCR prediction after NACT—one specific for ER + and another for ER- BC [36]. Similarly, Kallarackal et al. identified a three-gene signature for pCR in varying molecular subtypes of BC [37]. Additional studies focused on either *ER* + and/or *HER2* + BC [38–40], with a few focusing explicitly on TNBC. Notably, Zhao et al. introduced a NACT response probability score based on gene expression profiles in patients with *ER* + and TNBC cancer [22]. However, this signature involves more than 12,000 differentially expressed genes between pCR and RD with substantial issues in terms of transferability to clinical practice. Similarly, Pineda et al. developed an epigenetic score based on the methylation status of two genes (*FER3L* and *TRIP10*), which can predict the response to NACT in patients with TNBC [41]. While this signature is parsimonious, it was developed and validated using data from 24 and 30 patients, respectively, and needs additional testing on larger cohorts.

Our study's primary objective was to develop a gene signature predictive of pCR in TNBC patients undergoing NACT using pretreatment gene expression profiles obtained from standard tissue samples. To accomplish this, we used gene expression profiles from 369 pre-NACT TNBC samples, of which 223 samples were used for training and the remainder for testing. We leveraged our group's recent demonstration of the importance of incorporating biological constraints in predictive classifiers [9, 42] and used this approach to identify a subset of features predictive of NACT response. Specifically, we first designed a biological mechanism capturing the Notch signaling network by pairing genes known to be upregulated with those downregulated by Notch signaling. This mechanism was subsequently used as a biological constraint [9] to train a k-top scoring pairs (k-TSPs) model [16, 17] to select the maximum number of “Notch signaling” gene pairs that could distinguish patients who had RD from those with pCR in the training data. These pairs were then ranked using a regularized random forest (RRF) model trained on 100 bootstraps of the training data to select the most informative features. Finally, we selected the top five most frequent pairs as our final gene signature.

Our final Notch 5-TSPs signature comprises five gene pairs: *GARS*-*PDCD10*, *CCND1*-*FNDC3A*, *CMA1*-*PTHLH*, *F2R*-*KLF4*, and *PCDH7*-*TOX*, with each voting for a particular class (RD versus pCR) based on the relative ordering of the two genes. To fairly assess its performance, we applied the signature to two distinct testing sets with a total of 161 samples, resulting in AUCs of 0.76 and 0.87. In addition to this robust performance, several genes in our signature have known roles in BC pathophysiology. For instance, the gene pair GARS-PDCD10 is known to be involved in BC progression and chemosensitivity. In particular, while GARS is positively associated with BC progression and invasiveness through the activation of the mTOR signaling pathway [43], PDCD10 has been reported as a promoter of chemosensitivity in BC cell lines [44–46]. This is consistent with the decision rules of our Notch 5-TSPs signature, in which the *GARS*-*PDCD10* pair votes for RD if *GARS* is overexpressed relative to *PDCD10*. A similar pattern was also observed for the pair *F2R*-*KLF4*. In our signature, *F2R* (*PAR1*) overexpression relative to *KLF4* is predictive for RD, consistent with current evidence linking *F2R* overexpression to BC invasiveness and metastasis, especially in ER-negative tumors [47–50], while *KLF4* overexpression is a favorable prognostic factor, especially in patients with TNBC [51–53].

The biological consistency of our signature is further supported by its specificity for the triple negative subtype and its prognostic validity, demonstrated by its association with RFS in an independent TNBC cohort. Altogether, these findings support the validity of our approach in the development of predictive classifiers amenable to an effective translation to clinical practice. As such, classifiers developed using this method can be easily implemented as a clinical test using real-time polymerase chain reaction, even when trained on gene expression profiles from microarrays or RNA-seq [42].

Our study has certain limitations that must be taken into account. Despite achieving high accuracy in predicting pCR in TNBC, the dichotomous classification of response to therapy used in this work (pCR vs RD) is not the best option for clinical use. The residual cancer burden (RCB) classification system provides a more informative measure of response to therapy and patient prognosis. However, most datasets including BC patients undergoing NACT only provide pCR information and not RCB. Updating such datasets with the RCB variable and complete radiological information is critical for the implementation of models aimed at predicting the response to NACT and would improve the clinical validity of the resulting classifiers. Moreover, this study does not include patients treated with immune checkpoint inhibitors, which were recently approved for TNBC in the neoadjuvant setting. Therefore, the predictive power of our signature may differ in patients receiving such agents. Finally, the selection of genes based on existing knowledge of the Notch signaling pathway may have overlooked other critical genes involved in pCR prediction in TNBC.

Despite these limitations, our approach's application to the design of clinically useful classifiers can significantly enhance both test performance and robustness. Further research is necessary to validate our signature's clinical utility and generalizability in larger, prospective studies within the context of current neoadjuvant treatment options.

### Supplementary Information

**Additional file 1: Table S1.** Summary of the datasets included in the analysis. All samples were taken from triplenegative breast cancer patients before initiating neoadjuvant chemotherapy. T taxane, A anthracycline, F fluorouracil, C cyclophosphamide, E epirubicin, FNA fine needle aspiration, NACT neoadjuvant chemotherapy. **Figure S1**. Prognostic value of the TSP signature in patients with triple-negative breast cancer in the METABRIC dataset. **A** Receiver operating characteristic (ROC) curve showing the performance of the Notch TSP signature in predicting relapse-free survival in triple-negative breast cancer patients (n = 157). **B** Kaplan‒Meier survival curves showing the association between the signature’s predictions and relapse-free survival in the same set of patients. **C** Kaplan‒Meier survival curves demonstrating the association of each individual pair and relapse-free survival in the same set of patients. ‘p’ denotes the log-rank p value.

**Additional file 2**. Table of the pairs of up- and down-regulated genes by Notch signaling. These pairs were used as biological constraints during the training of the k-top scoring pairs (k-TSPs) algorithm.

**Additional file 3.** Table of the selected gene pairs using the k-top scoring pairs (k-TSPs) algorithm.

## Data Availability

The datasets used in this study are publicly available in the Gene Expression Omnibus (GEO).
