# Supplementary material for: Notch-based gene signature for predicting the response to neoadjuvant chemotherapy in triple-negative breast cancer
Source: J Transl Med. 2023 Nov 15;21:811. doi: 10.1186/s12967-023-04713-3 (PMC10647131; doi:10.1186/s12967-023-04713-3)
Supplement: Supplementary file 3 — Additional file 3. Table of the selected gene pairs using the k-top scoring pairs (k-TSPs) algorithm. [file 12967_2023_4713_MOESM3_ESM.docx]

**Additional file 1**

**Additional file 1**

**Tables**

**Table S1. Summary of the datasets included in the analysis.** All samples were taken from triple-negative breast cancer patients before initiating neoadjuvant chemotherapy. T: taxane, A: anthracycline, F: fluorouracil, C: cyclophosphamide, E: epirubicin, FNA: fine needle aspiration, NACT: neoadjuvant chemotherapy.

| **Accession** | **Sample Type** | **NACT regimen** | **Technology** | **N of samples** |
| --- | --- | --- | --- | --- |
| **GSE25055** | FNA/core biopsy | T/A | GPL96 | 112 |
| **GSE25065** | FNA/core needle biopsy | T/A | GPL96 | 58 |
| **GSE140494** | core needle biopsy | TFAC | GPL570 | 22 |
| **GSE103668** | core needle biopsy | cisplatin - bevacizumab | GPL570 | 21 |
| **GSE20271** | FNA | FAC/FEC | GPL96 | 59 |
| **GSE32646** | core needle biopsy | TFAC | GPL570 | 26 |
| **GSE20194^*^** | FNA | TFAC | GPL96 | 71 |

^* Only used for final independent evaluation of the signature performance.^

**Additional file 1**

**Figures**

**Figure S1. Prognostic value of the TSP signature in patients with triple-negative breast cancer in the METABRIC dataset.** A) Receiver operating characteristic (ROC) curve showing the performance of the Notch TSP signature in predicting relapse-free survival in triple-negative breast cancer patients (n=157). B) Kaplan‒Meier survival curves showing the association between the signature’s predictions and relapse-free survival in the same set of patients. C) Kaplan‒Meier survival curves demonstrating the association of each individual pair and relapse-free survival in the same set of patients. ‘p’ denotes the log-rank p value.
